# Impact of diabetes diagnosis on dental care utilization: evidence from Finland

**DOI:** 10.1186/s13561-023-00440-z

**Published:** 2023-05-02

**Authors:** Mikko Nurminen, Hanna Rättö

**Affiliations:** Research Department, The Social Insurance Institution of Finland, P.O. Box 450, Helsinki, 00056 Finland

**Keywords:** Diabetes, Dental care, Difference-in-differences, Finland

## Abstract

**Background:**

Poor oral health is associated with many chronic diseases, including diabetes. As diabetes can worsen oral health and vice versa, care guidelines recommend that patients with diabetes maintain good oral health and have regular dental checkups. We analyzed the impact of receiving an initial type 2 diabetes diagnosis on dental care utilization.

**Methods:**

We used register data on residents aged over 25 in the city of Oulu, Finland, covering the years 2013–2018. We used the difference-in-differences method and individuals with no diabetes diagnosis as control group. As robustness checks, we used propensity score matching and constructed an alternative control group from patients that received the same diagnosis a few years apart.

**Results:**

Despite the guideline recommendations, we found that receiving a diabetes diagnosis did not increase the probability for dental care visits in a two-year follow-up. The findings remained similar for both high-income and low-income persons.

**Conclusions:**

The finding is concerning in terms of diabetes management and oral health. Further research is needed on the reasons behind the lack of response to guidelines.

**Supplementary Information:**

The online version contains supplementary material available at 10.1186/s13561-023-00440-z.

## Background

In 2021, the number of adults living around the world with diabetes was 537 million, and their number is predicted to increase to 643 million by 2030 [1]. Diabetes increases the risk of complications in several organ systems, including the risk of oral diseases [2–4]. High glucose level causes decreased salivation and gum inflammation (gingivitis) [5–7], which may progress into periodontitis and eventually tooth loss. It has been estimated that the global prevalence of periodontitis is 10–15% [2, 8], and the estimated risk is two to three-fold higher for diabetics [2]. The link may also be bidirectional: poor oral health may promote diabetes [2, 9, 10] and treatment of periodontal diseases may improve glycemic control [11, 12]. Poor oral health is associated with decreased general health and quality of life [13–15]. Despite this, survey studies have found that diabetic patients’ knowledge on the importance of oral health is lacking [5, 7, 16]. Diabetic patients typically also have lower dental care utilization than non-diabetic persons [5, 17, 18]. Care guidelines [19–22] recommend that those with diabetes should practice good oral hygiene and seek regular oral health check-ups.

In this study, we analyzed the impact of receiving an initial type 2 diabetes diagnosis on dental care utilization. We leveraged comprehensive register data for residents of a Finnish city from the period 2013–2018. We used the difference-in-differences (DiD) method and compared those who received a diagnosis to a non-diagnosed population. We constructed robustness checks by using two alternative control groups. First we used propensity score matching (PSM). Second, to take into account potential unobserved heterogeneity [23, 24], we constructed a control group from those who also received a diagnosis but a few years later in the sample period. We analyzed visits to dentists and dental hygienists separately.

We contribute to the literature that has studied dental care utilization among diabetic patients. This literature has mainly focused on estimating the association between having diabetes and dental care utilization [5, 7, 17, 18, 25, 26]. In this paper, we directly estimated the impact of receiving a diabetes diagnosis on dental care utilization. Furthermore, the utilization of detailed register data allowed us to comprehensively assess realized dental care visits across both public and private providers. More generally, we also contribute to the literature on patient health-related decision making and behavior responses to diabetes and other chronic conditions [27–33]. The results from this literature has been mixed in terms of health behavior changes, although some evidence of increased medical spending and physician visits in response to diabetes diagnosis has been found [30–32]. By studying the impact on dental care utilization, we provide important evidence of behavioral (non-)responses to a vital aspect of complication management in diabetes.

## Methods

### Institutional background

The Finnish Current Care Guidelines recommend regular dental care visits for diabetics [22]. According to the guidelines, a physician should refer a diabetic patient to dental care. The dentist should then assess the individual needs for dental treatment intervals. For patients with diabetes the treatment intervals should be a maximum of six months. For smokers and those with moderate or severe periodontal disease, the examination and periodontal maintenance interval should be 3-4 months.

In Finland, medical services are organized in three separate co-existing sectors: public sector, private sector, and occupational health services (OHS) [34]. Diabetes can be diagnosed in each of the three sectors. Anyone can visit a medical professional in public and private sectors. However, OHS are available only for the employed population. Employers in Finland have a statutory obligation to provide preventive OHS for their employees, and many employers also offer curative primary care services. In addition, students form their own small health care scheme, but we exclude this sector from this study.

Dental care services are typically provided only through the public and private sectors. Public health care consists of universal coverage for all Finnish residents and is financed through taxes and co-payments. Primary care services are organized by municipalities, while specialized care services are organized by larger hospital districts. In dental care, dentist visits typically precede dental hygienists treatment. Depending on the health care service and the organizing municipality, the services are free of charge or carry a small fixed co-payment. For example, the co-payment ceiling for a visit to a dentist was 13.30 euros in 2022 [35].

The Private sector is a free-market-based fee-for-service system. Still, part of the payment is covered by the National Health Insurance (NHI) system. The NHI reimbursements are fixed amounts. In 2021, the average price for a dentist’s examination was 67 euros and the average NHI reimbursement for this procedure was 14 euros [36]. Since 2015, the NHI has reimbursed private dental service fees every other calendar year, unless the health of the patient necessitates annual reimbursements. Typically, the waiting times in the private sector have been shorter than in the public sector. Thus, the private sector has historically had a strong role in dental care provision [37]. The population shares of the attendees between public and private dental care have been roughly of similar sizes [37, 38], but those with higher income and socioeconomic status are more likely to utilize private care [37].

### Study population

The study population was individuals aged 25 and over living in the city of Oulu, Finland. With an approximate population of 200,000, Oulu is the fifth largest city in Finland. The data was collected from several registers for the period 2013–2018. We focused on individuals aged 25 and over and excluded individuals who had been students for at least one year during the study period, as we had no comprehensive data on the student health care system. Additionally, we restricted the sample to those who had the city of Oulu as their municipality of residence throughout the period 2013–2018.

### Diabetes diagnoses

Information on type 2 diabetes diagnoses for the study population came from public primary care, public primary and specialized care, OHS, and special medicine reimbursements. Private health care was not used to identify diagnoses as the register data on private health care did not include information on diagnoses.

Public primary care utilization was gathered from two registers: the register of the city of Oulu and the register of Primary Health Care Visits, maintained by the Finnish Institute for Health and Welfare. These registers contain information on the dates of visits and the received diagnoses. Both ICPC-2 and ICD10 coding systems are used in recording the information. Data on public specialized care utilization was gathered from the Care Register of Health Care maintained by the Finnish Institute for Health and Welfare. This register also includes ICD10-coded information on the dates of the visits and the diagnoses received.

OHS visits were gathered from the registers of four separate private providers: Mehiläinen, Terveystalo, Attendo, and Työterveys Virta. Together these registers account for over 90% of all OHS visits in the city of Oulu [39]. They record the dates of the visits and the received diagnoses using ICD10 coding.

The special reimbursement register, maintained by the Social Insurance Institution of Finland, contains information on all entitlements to special reimbursements for medicine expenses. Patients with diabetes can apply for a special, disease based NHI reimbursement. A medical certificate from a physician is needed in the application process. The register records the diagnosis associated with the special reimbursement using ICD10 coding. The register also records the month and year of the beginning and end of the entitlement.

Using the information in all these registers, we identified the earliest date (if any) of diabetes diagnosis for the study population. This was done to ensure that the earliest date of diagnosis was plausibly identified. From the ICPC-2 codes, we included all diagnoses in the "T90" category. From the ICD10 codes, we included all diagnoses in the "E11-E14" categories. Finally, we aggregated the initial diagnosis dates to individual-biannual level. We followed individuals for two years before and after the diagnosis. This, in practice, meant that the diagnosis had to have been received between 2015 and the second half of 2016.

### Dental care utilization

Overall dental care utilization in the period of 2013–2018 was identified from public and private health care utilization. Public visits to dental care were gathered from the register of the city of Oulu, while private dental care visits were gathered from the registers of the Social Insurance Institution of Finland. Private dental care included all visits and procedures that were reimbursed under the NHI scheme. We separately identified visits to dentists and dental hygienists. We used a binary variable to indicate whether an individual had visited a dentist or a dental hygienist during a biannual period.

### Other covariates

Age and sex were gathered from the registers of the Social Insurance Institution of Finland. In the regression estimations, age was grouped to bins (25-34, 35-44, 45-54, 55-64, 65-74, and over 74).

We measured socioeconomic background by education, occupational class, and annual taxable income. Socioeconomic background and occupational class information was retrieved from Statistics Finland, while information on annual taxable income was retrieved from the Finnish Tax Administration. Education was divided into four categories: upper tertiary, lower tertiary, secondary, and basic. Occupational class was divided into seven categories: upper-level non-manual, lower-level non-manual, manual, self-employed, unemployed, retired, and other (including unknown). Annual income included income from salaries and capital. The previous literature on dental care utilization has documented that income is positively correlated with the likelihood of dental care visits [37, 40, 41]. Thus, we also divided individuals into four groups by quartiles based on their total income in the first pre-diagnosis year.

As a proxy for chronic morbidities, we used the annual number of entitlements to special medicine reimbursements. In the regression estimations we binned these numbers to three groups: 1, 2-3, and over 3.

### Empirical framework

We estimated the effect of receiving an initial diabetes diagnosis on dental care utilization by comparing those who received a diagnosis to those who did not in a DiD framework. In effect, we examined the differences in the outcomes before and after receiving the initial diagnosis between the two groups. The causal inference through this framework relies on the assumption that the variation in dental care utilization is unrelated to the timing of receiving a diabetes diagnosis. In other words, absent of the diagnosis, the outcomes would run a parallel trend to that in the non-diagnosed population. This assumption would be violated if some uncontrollable characteristics of individuals should affect both the probability of receiving a diagnosis and dental care utilization.

As the non-diagnosed individuals did not have an initial diagnosis date, we used randomization to assign them one. We followed both diagnosed and non-diagnosed individuals for two years before and two years after the initial diagnosis. Formally, we estimated the following DiD specification:

1 $$\begin{aligned} y_{it}= & {} \alpha + \delta Diabetes_{i} + \lambda After_{it} + \beta {Diabetes_{i}}\times {After_{it}} \nonumber \\{} & {} + \theta X_{it} + \gamma _{t} + \epsilon _{it}. \end{aligned}$$

Here $y_{it}$ denotes a binary outcome whether individual *i* had visited a dentist at period *t*. Time was measured at half-year level. $\alpha$ is a constant, $Diabetes_{i}$ is an indicator for individuals diagnosed with diabetes, $After_{it}$ is an indicator equal to one for post-diagnosis periods, and $Diabetes_{i}\times {After_{it}}$ is the interaction of these two indicators. $X_{it}$ is a set of individual-level characteristics, $\gamma _t$ is time fixed effects, and $\epsilon _{it}$ is an error term. $\lambda$ shows the difference in the outcome levels between the two groups in the pre-diagnosis period, $\lambda$ shows the change in the outcome for the non-diagnosed group, and $\beta$ is the parameter of interest and shows the average effect of receiving a diabetes diagnosis on individuals’ outcomes. We estimated Equation 1 by ordinary least squares. We clustered standard errors at the individual level.

To test the parallel trends assumption, we plotted the trends in the outcomes for the two groups before and after the period of receiving the initial diagnosis. The absence of differential changes in the outcome trends of the groups in the pre-diagnosis periods alleviates concerns that the non-diagnosed group would not provide an appropriate counterfactual of the trend. We also estimated specifications where we controlled for individual fixed effects (FE) $\phi _{i}$:

2 $$\begin{aligned} y_{it}= & {} \alpha + \lambda After_{it} + \beta {Diabetes_{i}}\times {After_{it}} \nonumber \\{} & {} + \theta X_{it} + \gamma _{t} + \phi _{i} + \epsilon _{it}. \end{aligned}$$

The benefit of adding these fixed effects is that they control for time-invariant unobserved heterogeneity across individuals.

As a robustness test and to explicitly ensure that the two groups were similar to each other in observable characteristics, we combined the DiD approach with PSM. Using demographic and socioeconomic characteristics from the pre-diagnosis year 2013 (first year in the sample data), we matched each eventually diagnosed person with a unique control. This enabled us to assign each matched non-diagnosed person a treatment date equal to their diagnosed counterpart. Additional file 2 presents these estimation results for this sample.

However, it could still be that, even after controlling for individual characteristics and fixed effects and using PSM to balance the comparison groups with respect to these characteristics, there are some time-varying unobserved variables that may bias the estimations. For example, changes in lifestyle related habits, such as health behavior, may affect both the probability of diabetes and the trend in dental care utilization. Thus, as a robustness check, we additionally estimated the model using an alternative control group. Following the previous literature [23, 24, 42, 43], we constructed the control group from individuals who similarly received a diabetes diagnosis but not until a few years later in the sample period. For these individuals, we assigned a "placebo" diagnosis made a few years earlier than the actual time of the initial diagnosis. The idea is that those in the sample who were diagnosed later are similar in potentially confounding unobservable characteristics to those who were diagnosed earlier. Here the plausibility of the identification assumption also relies on the notion that the timing of receiving a diagnosis is as good as random within the sample window across the diagnosed individuals. See Additional file 3 for a detailed description of this empirical setting and the estimation results.

### Descriptive statistics

Table 1 shows the descriptive statistics for the non-diagnosed and diagnosed group in the pre-diagnosis year 2013. The full sample depicts individuals who were residents of the city of Oulu during 2013–2018, had no student status, and had at least two years of observations before and after the initial diagnosis (or a randomly assigned diagnosis period for the non-diagnosed group). In the full sample, the overall numbers of non-diagnosed and diagnosed individuals were 30,583 and 1,271, respectively. The table shows that the diagnosed were slightly more likely to be males, to have lower educational attainment, to be retired, and to have lower income. The differences between the two groups were notable with respect to many of the characteristics. For comparison, the table also shows the statistics after matching. To improve similarity across the occupational classes between the groups, the PSM algorithm was forced to perform exact matching on this categorical variable. After the matching, the *P*-values showed no statistically significant differences in the covariates between the groups.

**Table 1:** Descriptive statistics for individual-level characteristics in year 2013

	Full sample		Matched sample		
	No diabetes	Diabetes	No diabetes	Diabetes	*P*-value
Sex (%)					
Male	48.58	57.99	57.91	57.99	0.979
Female	51.42	42.01	42.09	42.01	0.968
Mean age	50.07	58.82	58.85	58.82	0.457
Education (%)					
Upper tertiary	15.85	7.00	6.85	7.00	0.871
Lower tertiary	27.69	20.61	20.30	20.61	0.762
Secondary	38.36	41.62	43.59	41.62	0.310
Basic	18.09	30.76	29.27	30.76	0.492
Occupational class (%)					
Upper-level non-manual	18.56	8.65	8.65	8.65	1
Lower-level non-manual	24.99	16.05	16.05	16.05	1
Manual worker	13.96	12.27	12.27	12.27	1
Self-employed	5.26	4.01	4.01	4.01	1
Unemployed	7.82	9.21	9.21	9.21	1
Retired	27.70	48.47	48.47	48.47	1
Other	1.70	1.34	1.34	1.34	1
Mean income (euros)	34,278.16	29,482.07	29,395.11	29,482.07	0.190
Mean number of special					
medicine reimbursements	0.03	0.03	0.04	0.03	0.843
Number of individuals	30,583	1,271	1,271	1,271	

Notes: The *P*-values for the categorical variables (Sex, Education, and Occupational class) are separately calculated using logistic regressions. For the other variables, the *P*-values are obtained using two-sided T-tests. The matching algorithm performed exact matching on occupational class categories

Table 2 shows the descriptive statistics on dental care and dental hygienist visits. The values are calculated across the whole two-year pre-diagnosis period. A large fraction, 39% of the individuals who were eventually diagnosed, had no recorded dentist or dental hygienist visits. Similarly for the non-diagnosed group, the fraction of these individuals is significant, being approximately 33%. Among diagnosed individuals and non-diagnosed individuals, respectively, 60% and 65% had at least one dentist visit during the pre-diagnosis period. The mean number of dentist visits per individual was approximately 3 in both groups. Visits to dental hygienists were less frequent, approximately 0.2 per individual in both groups.

**Table 2:** Overall visits to dentists and dental hygienists in the pre-diagnosis period

	%		Mean	
	No diabetes	Diabetes	No diabetes	Diabetes
No visits	32.98	38.95		
Has dentist visits	65.31	60.19		
Has dental hygienist visits	16.88	13.06		
Number of visits			2.91	2.91
Number of dentist visits			2.67	2.72
Number of dental hygienist visits			0.24	0.20

Notes: The sample used is the full sample. The values are calculated across the two-year pre-diagnosis period

**Fig. 1:**
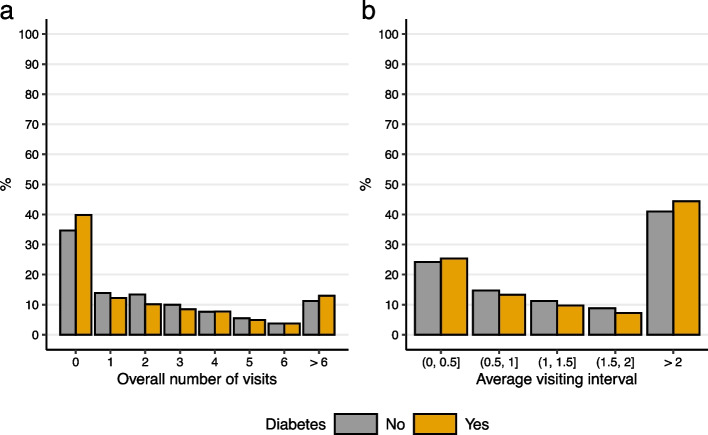
Distributions of dentist visits and visiting intervals in the pre-diagnosis period. The values are calculated from the two-year pre-diagnosis period. The Y-axis depicts the percentage of individuals. Average visiting interval depicts the average time between consecutive visits per person, measured in half-year intervals. Those who had one or no visits in the pre-diagnosis period were categorized with average visiting interval over two

To shed more light on the frequency of pre-diagnosis dentist visits, Fig. 1 plots the distributions of overall number of visits and average visiting intervals. For reference, Fig. 1 also plots the distributions of those who had not received a diagnosis. Approximately 20% of the diagnosed individuals had two or fewer visits in this period. The total fraction of diagnosed individuals who had annual visits was below 40%. A large fraction, over 40% of the diagnosed individuals, had over two years between dentist visits. The fraction of individuals who had on average between one and two years between visits was slightly under 20%.

## Results

### Descriptive results

**Fig. 2:**
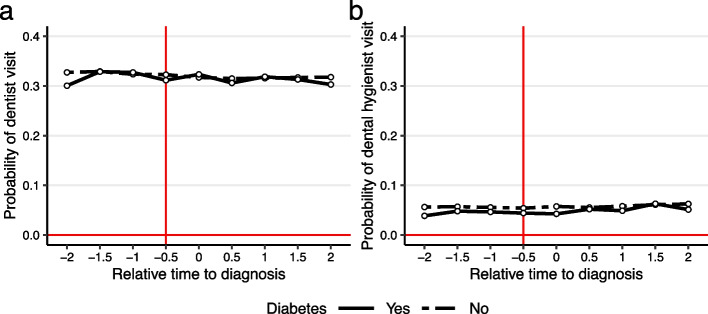
Probability of dentist and dental hygienist visits. Relative time is measured in half-years

Figure 2 displays the probabilities of dentist (Fig. 2a) and dental hygienist (Fig. 2b) visits around the time of the initial diagnosis measured at biannual intervals. For both groups, the diagnosed and non-diagnosed, the probabilities of dentist visits remained stable, at approximately 30% throughout the follow-up period. Similarly, no notable differences could be found for the probabilities of dental hygienist visits between the pre- and post-diagnosis periods. Crucially, Fig. 2 showed no differential pre-trends between the two groups, and thus the parallel trend assumption behind the DiD estimator was not violated. Additional file 2 Fig. B1 and Additional file 3 Fig. C2, respectively, show that the results are similar for the matched sample and the early versus later treated.

### DiD results

Table 3 shows the DiD estimates. The first column shows that, in line with the descriptive results above, receiving a diabetes diagnosis had virtually no impact on the probability for visiting a dentist. The coefficient estimate is close to zero (0.008), and quite precisely estimated. Using the 95% confidence intervals, effects larger than a 2%-point increase in the probability of dentist visit could be ruled out. The second column shows that the coefficient estimates did not change when adjusted for individual fixed effects. The third column shows the regression estimates for the probability of visiting a dental hygienist. As with dentist visits, no statistically significant impact was found for this outcome. The fourth column shows the robustness of the estimates to the inclusion of individual fixed effects.

**Table 3:** Effect of diabetes on dental care visits

	Outcome			
Variable	Dentist	Dentist	Dental hygienist	Dental hygienist
Diabetes	−0.014		−0.008$$^{**}$$	
	(0.009)		(0.004)	
After	0.003	−0.001	−0.002	0.000
	(0.004)	(0.003)	(0.002)	(0.002)
Diabetes$\times$After	0.008	0.006	0.004	0.004
	(0.008)	(0.008)	(0.004)	(0.004)
Individual FE	No	Yes	No	Yes
N	286,686	286,686	286,686	286,686

Notes: The sample used is the full sample. Each column is from a separate regression. The outcomes are binary variables. Additional controls included are time fixed effects, sex, age groups (25-34, 35-44, 45-54, 55-64, 65-74, and $>74$), log annual income, education, occupational class, and the grouped number of special medicine reimbursements (1, 2-3, and $>3$). The control for sex is omitted from the regressions that control for individual fixed effects. The standard errors are clustered at the individual level and are shown in parentheses. * *p* $<0.1$, ** *p* $<0.05$, *** *p* $< 0.01$

Table 4 shows the results for coefficients estimated separately for each income quartile. For both outcomes, and across all income quartiles, the DiD coefficient point estimates are statistically insignificant and close to zero. The results were not impacted by including individual fixed effects. However, it could also be that a higher income only impacts the likelihood of private dental care visits. Table  5 shows the results for the probability of private health care visits. As with the overall dental visiting probability, the point estimates were close to zero and statistically insignificant across all income quartiles.

**Table 4:** Effect of diabetes on dental care visits, by income quartile

Variable	Quartile 1	Quartile 1	Quartile 2	Quartile 2	Quartile 3	Quartile 3	Quartile 4	Quartile 4
Panel A. Dentist								
Diabetes	−0.029$$^{**}$$		0.012		−0.039$$^{**}$$		0.022	
	(0.014)		(0.017)		(0.020)		(0.022)	
After	−0.005	0.000	0.002	−0.006	0.003	−0.001	0.012$^{*}$	0.005
	(0.007)	(0.006)	(0.007)	(0.007)	(0.007)	(0.007)	(0.007)	(0.007)
Diabetes$\times$After	0.005	0.004	0.011	0.011	0.009	0.012	0.005	0.001
	(0.012)	(0.012)	(0.015)	(0.015)	(0.019)	(0.019)	(0.019)	(0.019)
Panel B. Dental hygienist								
Diabetes	−0.015$$^{***}$$		−0.015$$^{**}$$		0.008		0.005	
	(0.006)		(0.006)		(0.010)		(0.010)	
After	−0.005	0.000	0.003	0.003	−0.001	0.001	−0.005	−0.005
	(0.003)	(0.003)	(0.004)	(0.004)	(0.004)	(0.004)	(0.003)	(0.003)
Diabetes$\times$After	0.007	0.006	0.010	0.010	−0.003	−0.003	0.000	0.000
	(0.005)	(0.005)	(0.007)	(0.007)	(0.010)	(0.010)	(0.010)	(0.010)
Individual FE	No	Yes	No	Yes	No	Yes	No	Yes
N	71,676	71,676	71,667	71,667	71,667	71,667	71,676	71,676

Notes: The sample used is the full sample. Each panel and column combination is from a separate regression. The outcomes are binary variables. Income quartiles are calculated from the year preceding the treatment period. Additional controls included are time fixed effects, sex, age groups (25-34, 35-44, 45-54, 55-64, 65-74, and $>74$), log annual income, education, occupational class, and the grouped number of special medicine reimbursements (1, 2-3, and $>3$). The control for sex is omitted from the regressions that control for individual fixed effects. The standard errors are clustered at the individual level and are shown in parentheses. * *p* $<0.1$, ** *p* $<0.05$, *** *p* $< 0.01$

**Table 5:** Effect of diabetes on private dentist visits, by income quartile

Variable	Quartile 1	Quartile 1	Quartile 2	Quartile 2	Quartile 3	Quartile 3	Quartile 4	Quartile 4
Diabetes	−0.014		−0.006		−0.048$$^{***}$$		0.042$$^{*}$$	
	(0.010)		(0.015)		(0.017)		(0.023)	
After	−0.008$^{*}$	−0.005	0.008	0.001	−0.005	−0.006	0.010	0.003
	(0.005)	(0.004)	(0.006)	(0.005)	(0.006)	(0.005)	(0.007)	(0.006)
Diabetes$\times$After	0.005	0.002	−0.002	−0.007	0.003	0.002	0.002	−0.008
	(0.007)	(0.007)	(0.010)	(0.010)	(0.013)	(0.013)	(0.017)	(0.017)
Individual FE	No	Yes	No	Yes	No	Yes	No	Yes
N	71,676	71,676	71,667	71,667	71,667	71,667	71,676	71,676

Notes: The sample used is the full sample. Each column is from a separate regression. The outcomes is a binary variable. Income quartiles are calculated from the year preceding the treatment period. Additional controls included are time fixed effects, sex, age groups (25-34, 35-44, 45-54, 55-64, 65-74, and $>74$), log annual income, education, occupational class, and the grouped number of special medicine reimbursements (1, 2-3, and $>3$). The control for sex is omitted from the regressions that control for individual fixed effects. The standard errors are clustered at the individual level and are shown in parentheses. * *p* $<0.1$, ** *p* $<0.05$, *** *p* $< 0.01$

### Robustness checks

#### Propensity score matching

Table 1 showed that the diagnosed and non-diagnosed individuals differed in terms of socioeconomic and demographic characteristics. To take this explicitly into account, we ran the regressions for the matched sample obtained by PSM. The results are shown in Additional file 2. Table B2 shows that the results remained similar and that the DiD coefficient was close to zero.

Table B3 shows the results by income quartiles. No significant increases for dentist visits were found. For the probability of a dental hygienist visit in the second income quartile, the point estimate without individual fixed effects was positive and statistically significant at the 10% level. Figure B3 shows a minor pre-trend for this income group, but no distinct trend changes after receiving the diagnosis. Table B4 shows that, for the third income quartile, the probability of visiting a private dentist increased slightly after receiving a diabetes diagnosis. In conclusion, the results from these robustness checks were in line with the DiD results above.

#### Early versus later diagnosed

In Additional file 3, we replicated the estimations using an alternative control group of individuals who received the diagnosis two and half years later than the individuals in the treatment group. Arguably, the later diagnosed group is likely to be more similar to the earlier diagnosed group in terms of unobserved characteristics such as lifestyle habits. Additionally, it is unlikely that receiving a diabetes diagnosis a few years later in the sample is systematically linked to these potentially biasing factors.

Table C3 shows the results for the DiD estimations. For dentist visits, the results remained intact. For dental hygienist visits, the increase in the probability was statistically significant at the 10% level. However, the magnitude of the estimate was small: only a 1.1%-point increase was seen. Table C4 shows the results for the different income quartiles. Except for the probability of a dental hygienist visit in the second income quartile, the results remained similar to the DiD results. The results for private dentist visits showed no evidence of increased probability for visits (Table C5). Overall, the results in Tables 3, 4 and 5 were robust to using this alternative control group.

## Discussion

We studied the impact of receiving a type 2 diabetes diagnosis on the utilization of dental care. Guidelines of diabetes care recommend good oral hygiene and regular dental care visits [22]. Using comprehensive register data, we found, however, that receiving a diabetes diagnosis had no effect on the probability of using the services of either dentists or dental hygienists. As studies have shown that dental care utilization is associated with the socioeconomic status of the individual [37, 40, 41], we also studied the impact by income quartile. The results remained similar when taking income into account: receiving a diabetes diagnosis did not increase the probability for dental care visits for low or high-income persons.

Our results are in line with the previous literature that has showed that the level of dental care utilization among patients with diabetes is low [5, 7, 17, 18, 25, 26]. In contrast, the literature studying the impact of receiving a diabetes diagnosis on health behavior other than dental care utilization has found some increase in the follow-up outpatient visits and medical spending [30–32]. However, the findings of this literature on, for example, weight loss has been mixed [27, 30–33]. Additionally, long-term effects on other health behavior such as smoking and alcohol consumption has been found to be limited [27, 33]. There could be several reasons behind the results of our study.

First, it could be that dental care utilization was already at an adequate level before receiving the diagnosis. However, this study found that 40% had no dentist visits during the two years preceding the diagnosis. Also, the fraction of diagnosed individuals that, on average, visited dentists annually was less than 40%. While dental health has been slowly improving in the Finnish adult population, the prevalence of caries and periodontitis is still considerable and oral health is below that of many other European countries and the US [44]. According to the Finnish national health survey, men still brush their teeth less frequently than women [45]. The proportion of those adults who considered their subjective oral health to be good was 59% in men and 79% in females [45]. These findings indicate that an adequate level of dental care utilization is an unlikely explanation for the results.

Second, oral health knowledge and literacy may be poor within this patient population. Also, promotion of oral health risks and dental checkups by diabetes care providers could be inadequate. Promotion of oral health by health care professionals for patients with diabetes has been recognized as a vital aspect of disease management [5, 19], especially since knowledge of the importance of oral health has been found to be lacking [5, 7, 16].

Third, it could be that the wait times to get into public dental services were too long for individuals to seek dental care. The percentage of patients with a non-urgent appointment at a public health center who did not receive an appointment within three weeks in the city of Oulu in October 2018 was 58% [46]. However, the follow-up period in this study was two years following the diagnosis, which should have adequately captured postponed visits.

Fourth, the responses may be larger only among high-risk individuals that this study did not capture. Marginal benefits to increased dental care utilization may be higher among individuals with strongly elevated blood sugar and other health markers such as elevated cholesterol levels and blood pressure. Related to this, it could also be that patients in the initial stages of diabetes do not prioritize dental care checkups and respond first to the more traditional recommendations such as weight management, exercise, and dietary restrictions.

The strength of this study was the comprehensive register data that was utilized. Furthermore, the additional robustness checks using PSM and creating the control group from individuals who received the same diagnosis but a few years later in the sample alleviated concerns related to unobserved heterogeneity and strengthened the robustness of the results. However, some limitations need to be acknowledged. The data used in the analysis was from one city, and it remains possible that, despite the universal health care in Finland, treatment practices are not representative for the whole country. Also, the data did not contain information on the oral health of the patients. Thus, analyzing the heterogeneity of the results by the patients’ pre-diagnosis oral health status was not possible. Finally, the analysis of the impact was limited to a two-year follow-up period. Although, in terms of the guideline recommendations, the response to dental care utilization would be expected to be seen in the short run, it is possible that the impact is realized only in the long run and not until the condition has worsened.

## Conclusion

This study examined the utilization of dental care services after receiving a type 2 diabetes diagnosis. Care guidelines recommend that patients with diabetes maintain good oral health and have regular dental checkups. However, the probability to visit a dentist or a dental hygienist was not impacted by receiving a diagnosis in a two-year follow-up period. Strengthening diabetes patients’ knowledge of the importance of dental care and improving coordination of care between diabetes care providers and dentists could improve dental care attendance. Also, better resource targeting to patients with chronic diseases could further improve attendance among this population. Finally, more research is needed to clarify guideline recommendations about the efficiency of regular dental check-ups as a preventative measure for diabetes related health complications.

## Supplementary Information

**Additional file 1.** Additional results.

**Additional file 2.** Propensity score matching.

**Additional file 3.** Early versus later diagnosed.

## Data Availability

Due to legal restrictions and the data protection regulations of the administrative sources providing individual-level register data, the authors do not have the permission to make sensitive personal data available. For access to data on health services in the City of Oulu, Finnish Institute for Health and Welfare data, and data of the Social Insurance Institution of Finland, interested parties may apply to the centralized data permit authority Findata (https://www.findata.fi/en/, accessed on 10 November 2022), info@findata.fi. Applications for permission to access data on education and occupational class may be submitted to Statistics Finland (https://www.stat.fi/tup/mikroaineistot/index_en.html, accessed on 10 November 2022), tutkijapalvelut@stat.fi. Data on taxable income is available by application to the Finnish Tax Administration, verohallinto@vero.fi, P.O. Box 325, 00052 VERO.

## Abbreviations

DiD: Difference-in-differences
FE: Fixed effects
PSM: Propensity score matching
