# Supplementary material for: Impact of diabetes diagnosis on dental care utilization: evidence from Finland
Source: Health Econ Rev. 2023 May 2;13:26. doi: 10.1186/s13561-023-00440-z (PMC10152714; doi:10.1186/s13561-023-00440-z)
Supplement: Supplementary file 1 — Additional file 1. Additional results. [file 13561_2023_440_MOESM1_ESM.pdf]

Additional file 1: Additional results

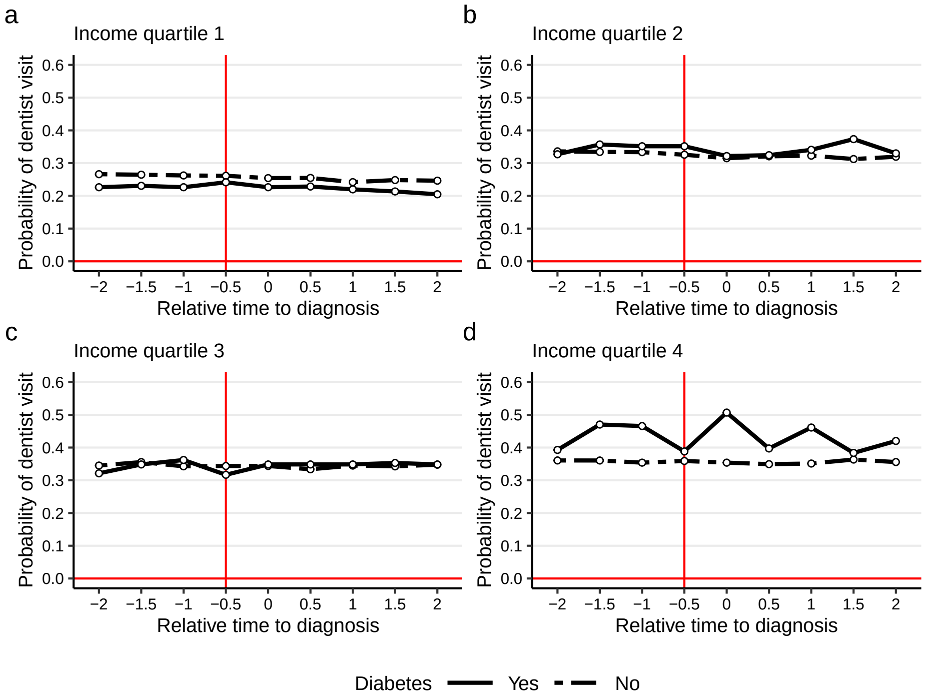

Figure A1: Probability of dentist visit, by income quartile. Quartile 1 is the lowest income quartile and quartile 4 is the highest income quartile. Relative time is measured in half-years.

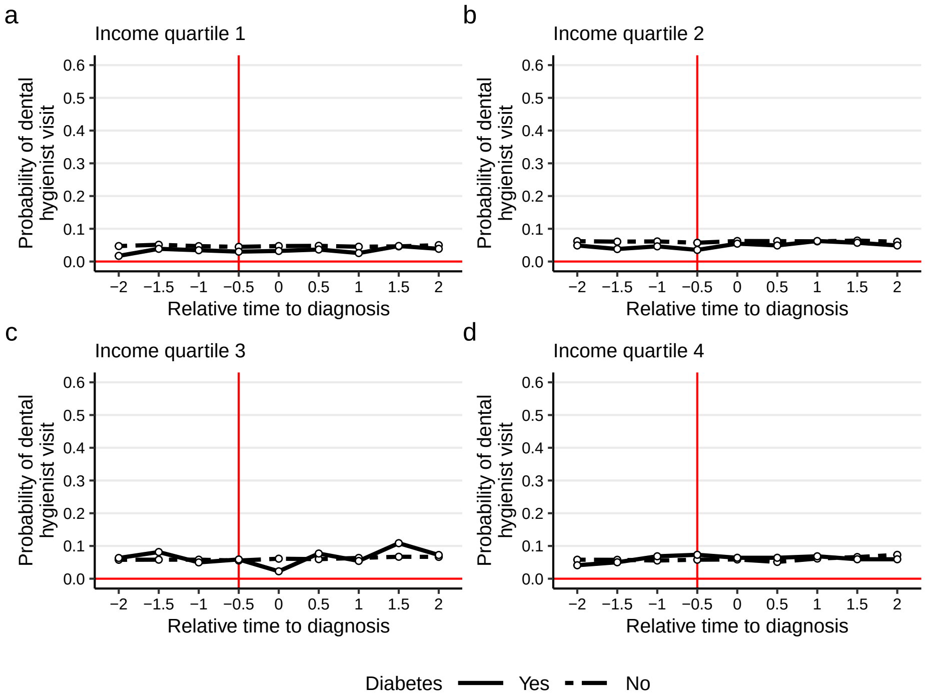

Figure A2: Probability of dental hygienist visit, by income quartile. Quartile 1 is the lowest income quartile and quartile 4 is the highest income quartile. Relative time is measured in half-years.
