# Supplementary material for: Impact of diabetes diagnosis on dental care utilization: evidence from Finland
Source: Health Econ Rev. 2023 May 2;13:26. doi: 10.1186/s13561-023-00440-z (PMC10152714; doi:10.1186/s13561-023-00440-z)
Supplement: Supplementary file 2 — Additional file 2. Propensity score matching. [file 13561_2023_440_MOESM2_ESM.pdf]

Additional file 2: Propensity score matching

Table B1: Overall visits to dentists and dental hygienists in the pre-diagnosis period

	%		Mean	
	No diabetes	Diabetes	No diabetes	Diabetes
No visits	36.27	38.95		
Has dentist visits	62.08	60.19		
Has dental hygienist visits	15.26	13.06		
Number of visits			2.94	2.91
Number of dentist visits			2.71	2.72
Number of dental hygienist visits			0.23	0.20

Notes: The sample used is the matched sample. The values are calculated across the two-year pre-diagnosis period.

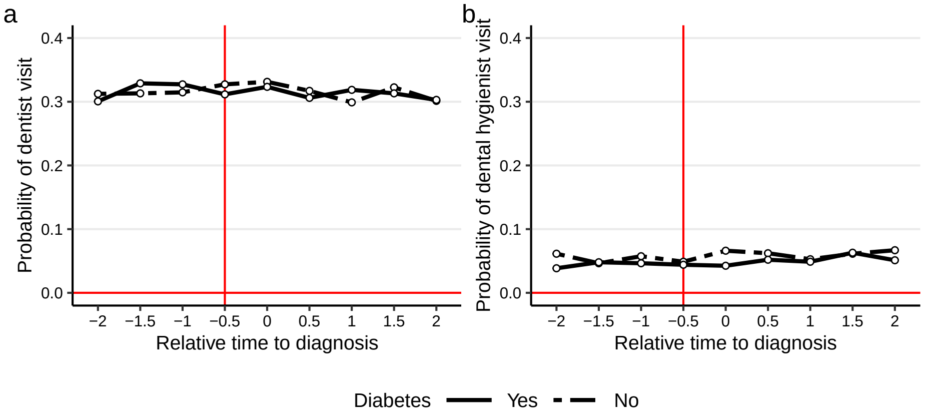

Figure B1: Probability of dentist and dental hygienist visits. Relative time is measured in half-years.

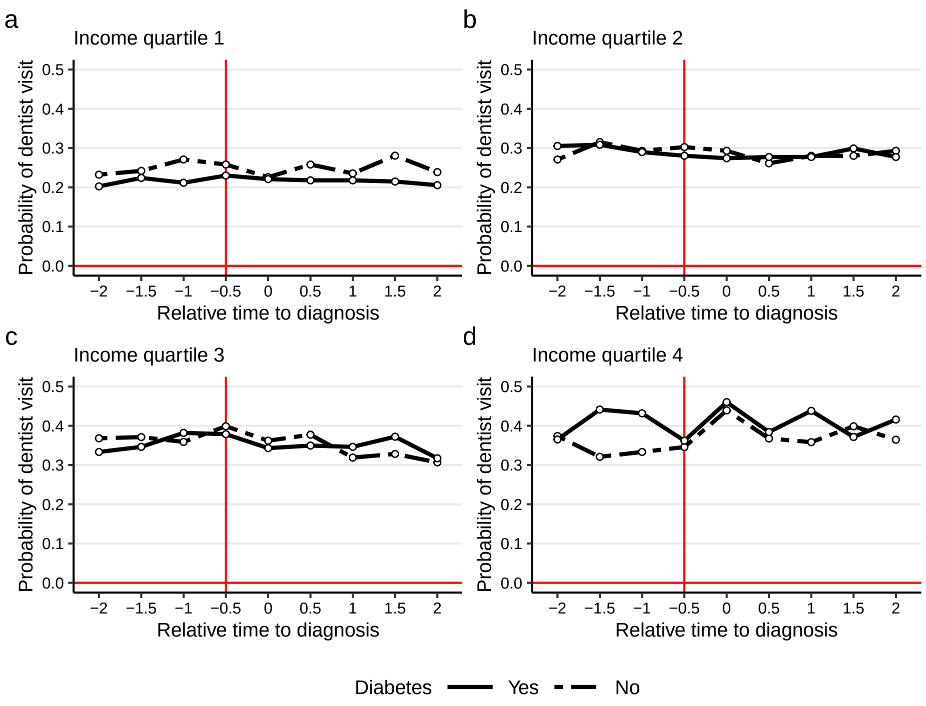

Figure B2: Probability of dentist visit, by income quartile. Quartile 1 is the lowest income quartile and quartile 4 is the highest income quartile. Relative time is measured in half-years.

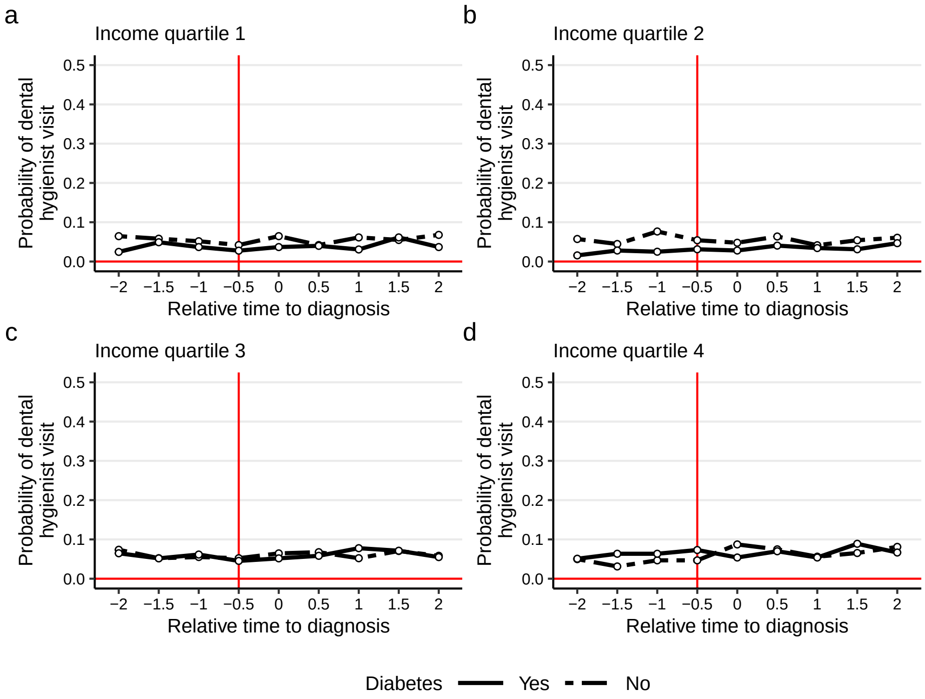

Figure B3: Probability of dental hygienist visit, by income quartile. Quartile 1 is the lowest income quartile and quartile 4 is the highest income quartile. Relative time is measured in half-years.

Table B2: Effect of diabetes on dental care visits

Variable	Outcome			
	Dentist	Dentist	Dental hygienist	Dental hygienist
Diabetes	−0.001		−0.010*	
	(0.012)		(0.005)	
After	0.025*	0.008	0.009	0.003
	(0.014)	(0.013)	(0.007)	(0.006)
Diabetes×After	0.003	0.001	−0.001	−0.001
	(0.011)	(0.011)	(0.005)	(0.005)
Individual FE	No	Yes	No	Yes
N	22,878	22,878	22,878	22,878

Notes: The sample used is the matched sample. Each column is from a separate regression. The outcomes are binary variables. Additional controls included are time fixed effects, sex, age groups (25-34, 35-44, 45-54, 55-64, 65-74, and > 74), log annual income, education, occupational class, and the grouped number of special medicine reimbursements (1, 2-3, and > 3). The control for sex is omitted from the regressions that control for individual fixed effects. The standard errors are clustered at the individual level and are shown in parentheses. * $p < 0.1$, ** $p < 0.05$, *** $p < 0.01$.

Table B3: Effect of diabetes on dental care visits, by income quartile

Variable	Quartile 1	Quartile 1	Quartile 2	Quartile 2	Quartile 3	Quartile 3	Quartile 4	Quartile 4
Panel A. Dentist								
Diabetes	−0.043*		0.005		−0.012		0.043*	
	(0.023)		(0.025)		(0.025)		(0.024)	
After	−0.024	−0.036	0.006	−0.008	0.024	−0.029	0.112***	0.105***
	(0.027)	(0.024)	(0.028)	(0.025)	(0.029)	(0.026)	(0.027)	(0.027)
Diabetes×After	−0.001	0.000	0.010	0.006	0.025	0.032	−0.027	−0.038*
	(0.021)	(0.021)	(0.022)	(0.021)	(0.022)	(0.021)	(0.023)	(0.023)
Panel B. Dental hygienist								
Diabetes	−0.021*		−0.034***		−0.004		0.018	
	(0.011)		(0.010)		(0.010)		(0.012)	
After	0.006	0.008	−0.002	−0.014	0.006	0.001	0.027*	0.016
	(0.012)	(0.012)	(0.013)	(0.012)	(0.014)	(0.014)	(0.015)	(0.013)
Diabetes×After	0.003	0.002	0.017*	0.016	−0.003	0.002	−0.023**	−0.022*
	(0.009)	(0.009)	(0.010)	(0.010)	(0.012)	(0.012)	(0.012)	(0.012)
Individual FE	No	Yes	No	Yes	No	Yes	No	Yes
N	5,724	5,724	5,715	5,715	5,715	5,715	5,724	5,724

Notes: The sample used is the matched sample. Each panel and column combination is from a separate regression. The outcomes are binary variables. Income quartiles are calculated from the year preceding the treatment period. Additional controls included are time fixed effects, sex, age groups (25-34, 35-44, 45-54, 55-64, 65-74, and > 74), log annual income, education, occupational class, and the grouped number of special medicine reimbursements (1, 2-3, and > 3). The control for sex is omitted from the regressions that control for individual fixed effects. The standard errors are clustered at the individual level and are shown in parentheses. * $p < 0.1$, ** $p < 0.05$, *** $p < 0.01$.

Table B4: Effect of diabetes on private dentist visits, by income quartile

Variable	Quartile 1	Quartile 1	Quartile 2	Quartile 2	Quartile 3	Quartile 3	Quartile 4	Quartile 4
Diabetes	−0.015		−0.001		−0.043*		0.025	
	(0.015)		(0.021)		(0.023)		(0.024)	
After	−0.001	−0.009	0.011	0.010	0.010	−0.047**	0.081***	0.068***
	(0.017)	(0.013)	(0.023)	(0.018)	(0.024)	(0.019)	(0.025)	(0.022)
Diabetes×After	0.002	0.001	−0.022	−0.021	0.031*	0.036**	−0.015	−0.024
	(0.011)	(0.011)	(0.015)	(0.015)	(0.017)	(0.016)	(0.019)	(0.018)
Individual FE	No	Yes	No	Yes	No	Yes	No	Yes
N	5,724	5,724	5,715	5,715	5,715	5,715	5,724	5,724

Notes: The sample used is the matched sample. Each column is from a separate regression. The outcome is a binary variable. Income quartiles are calculated from the year preceding the treatment period. Additional controls included are time fixed effects, sex, age groups (25-34, 35-44, 45-54, 55-64, 65-74, and > 74), log annual income, education, occupational class, and the grouped number of special medicine reimbursements (1, 2-3, and > 3). The control for sex is omitted from the regressions that control for individual fixed effects. The standard errors are clustered at the individual level and are shown in parentheses. * $p < 0.1$, ** $p < 0.05$, *** $p < 0.01$.
