# Supplementary material for: Impact of diabetes diagnosis on dental care utilization: evidence from Finland
Source: Health Econ Rev. 2023 May 2;13:26. doi: 10.1186/s13561-023-00440-z (PMC10152714; doi:10.1186/s13561-023-00440-z)
Supplement: Supplementary file 3 — Additional file 3. Early versus later diagnosed. [file 13561_2023_440_MOESM3_ESM.pdf]

Additional file 3: Early versus later diagnosed

The general idea is to overcome the potential selection bias and unobserved heterogeneity of those who receive a diabetes diagnosis. Comparing those who received the initial diabetes diagnosis earlier in the sample to those who received the initial diabetes diagnosis later in the sample, arguably increases the plausibility that the two groups are more similar in unobservable factors. This research design exploits the potential randomness of the timing of the initial diabetes diagnosis within a short period of time in the sample.

We formed the treatment group from those who received the initial diabetes diagnosis in 2015 or in the first half of 2016. We formed the control group from those who received the initial diagnosis in the second half of 2017 or in 2018. This ensured that there were at least two and half years between the initial diagnoses of the treatment and control groups. After this, for the control group, a "placebo" diagnosis was assigned to have been made two and half years earlier than the actual diagnosis. The trade-off in the length of this window is the similarity of the treatment and control groups and the length of time that the outcomes can be tracked following the diagnosis. In practice, the length of the panel in our sample, 2013–2018, limited us in exploring with various window sizes. Fig C1 illustrates the empirical setting.

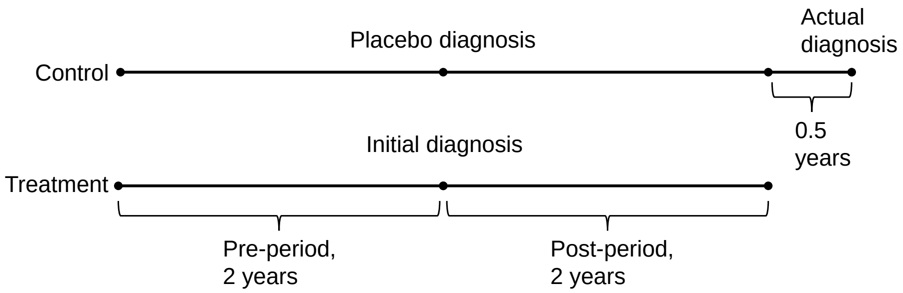

Figure C1: Illustration of treatment and control group formation

Table C1: Descriptive statistics for the covariates between early (treatment) and later (control) diagnosed individuals

	Control	Treatment	P-value
Sex (%)			
Male	53.03	58.84	0.368
Female	46.97	41.16	0.009
Mean age	58.63	58.93	0.271
Education (%)			
Upper tertiary	6.99	6.66	0.697
Lower tertiary	23.67	20.63	0.197
Secondary	39.98	42.58	0.733
Basic	29.36	30.13	0.109
Occupational class (%)			
Upper-level non-manual	8.11	8.30	0.864
Lower-level non-manual	15.38	16.27	0.367
Manual worker	10.34	12.55	0.431
Self-employed	4.94	4.48	0.513
Unemployed	10.25	9.50	0.468
Retired	49.58	47.49	0.446
Other	1.40	1.42	0.917
Mean income (euros)	27,884.29	29,541.50	0.076
Mean number of special medicine reimbursements	0.03	0.03	0.983
Number of individuals	1,073	916	

Notes: The P-values for the categorical variables (Sex, Education, and Occupational class) are separately calculated using logistic regressions. For the other variables, the P-values are obtained using two-sided T-tests.

Table C2: Overall visits to dentists and dental hygienists in the pre-treatment period

	%		Mean	
	Control	Treatment	Control	Treatment
No visits	37.00	38.54		
Has dentist visits	62.07	60.70		
Has dental hygienist visits	15.38	12.55		
Number of visits			3.00	2.98
Number of dentist visits			2.76	2.80
Number of dental hygienist visits			0.24	0.19

Notes: The sample used is the early versus later treated sample. The values are calculated from the whole pre-treatment period.

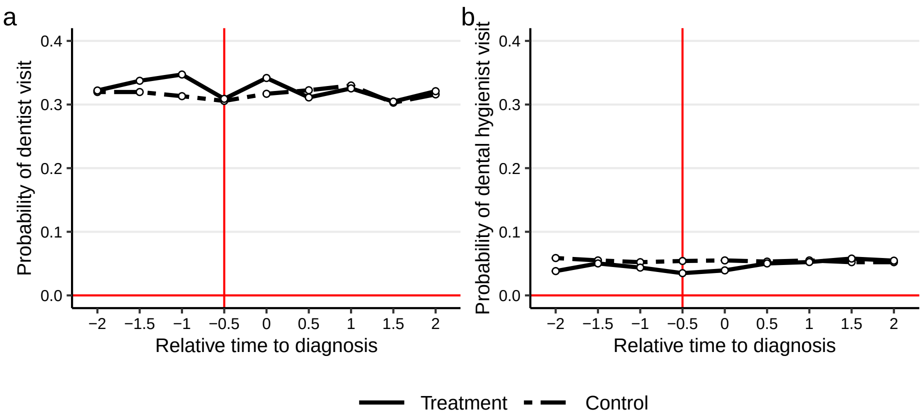

Figure C2: Probability of dentist and dental hygienist visits. Relative time is measured in half-years.

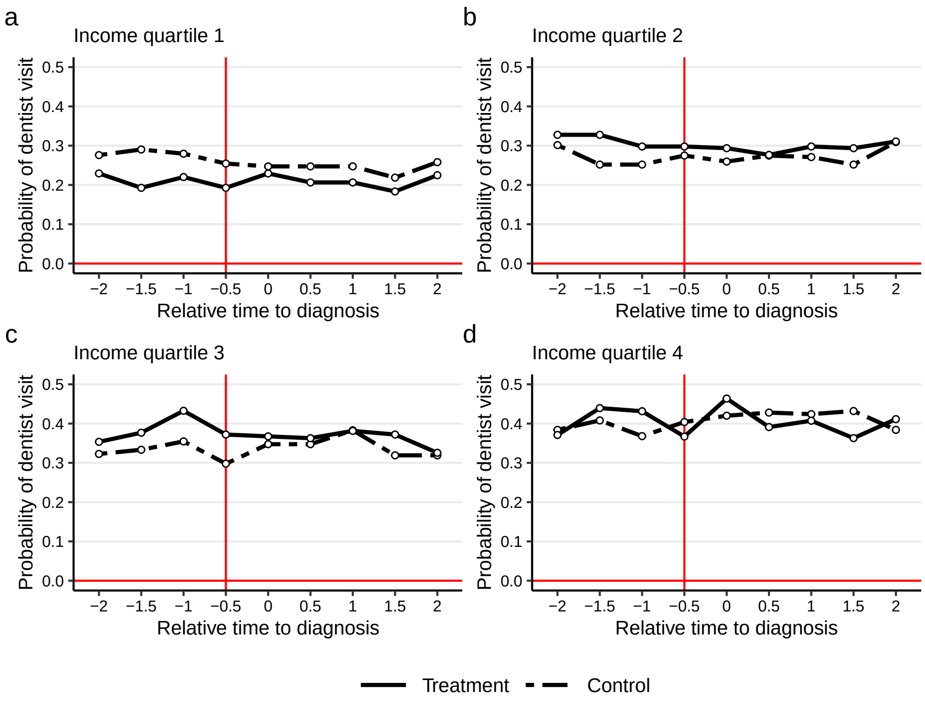

Figure C3: Probability of dentist visit, by income quartile. Quartile 1 is the lowest income quartile and quartile 4 is the highest income quartile. Relative time is measured in half-years.

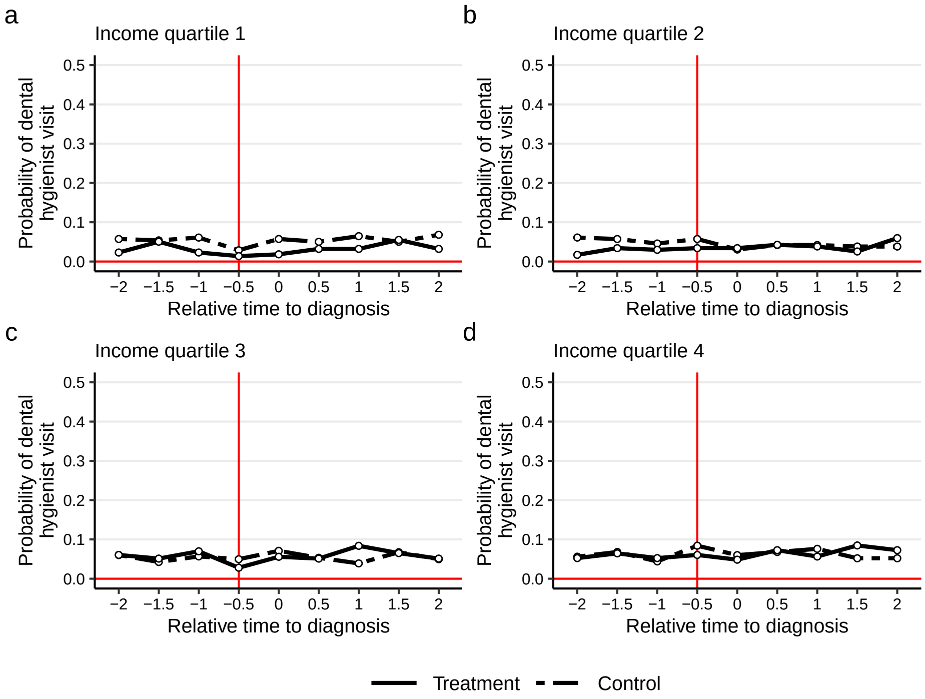

Figure C4: Probability of dental hygienist visit, by income quartile. Quartile 1 is the lowest income quartile and quartile 4 is the highest income quartile. Relative time is measured in half-years.

Table C3: Effect of diabetes on dental care visits

	Outcome			
Variable	Dentist	Dentist	Dental hygienist	Dental hygienist
Treatment	0.015		-0.012^{**}	
	(0.014)		(0.006)	
After	0.007	0.007	-0.002	0.001
	(0.017)	(0.015)	(0.008)	(0.007)
Treatment×After	-0.007	-0.008	0.011^{*}	0.011^{*}
	(0.013)	(0.012)	(0.006)	(0.006)
Individual FE	No	Yes	No	Yes
N	17,901	17,901	17,901	17,901

Notes: The sample used is the early versus later treated sample. Each column is from a separate regression. The outcomes are binary variables. Additional controls included are time fixed effects, sex, age groups (25-34, 35-44, 45-54, 55-64, 65-74, and > 74), log annual income, education, occupational class, and the grouped number of special medicine reimbursements (1, 2-3, and > 3). The control for sex is omitted from the regressions that control for individual fixed effects. The standard errors are clustered at the individual level and are shown in parentheses. * $p < 0.1$, ** $p < 0.05$, *** $p < 0.01$.

Table C4: Effect of diabetes on dental care visits, by income quartile

Variable	Quartile 1	Quartile 1	Quartile 2	Quartile 2	Quartile 3	Quartile 3	Quartile 4	Quartile 4
Panel A. Dentist								
Treatment	−0.058**		0.053*		0.056**		0.026	
	(0.028)		(0.028)		(0.028)		(0.027)	
After	−0.038	−0.017	−0.028	−0.021	0.011	0.010	0.102***	0.057*
	(0.033)	(0.028)	(0.033)	(0.030)	(0.035)	(0.032)	(0.035)	(0.033)
Treatment×After	0.025	0.031	−0.014	−0.017	−0.026	−0.015	−0.025	−0.029
	(0.024)	(0.024)	(0.024)	(0.023)	(0.028)	(0.027)	(0.026)	(0.026)
Panel B. Dental hygienist								
Treatment	−0.022*		−0.024**		0.003		−0.002	
	(0.011)		(0.011)		(0.012)		(0.014)	
After	0.013	0.025*	−0.029*	−0.023	0.023	0.027*	−0.015	−0.026*
	(0.013)	(0.014)	(0.016)	(0.015)	(0.017)	(0.014)	(0.016)	(0.014)
Treatment×After	−0.003	−0.004	0.030**	0.031**	0.007	0.009	0.007	0.008
	(0.011)	(0.011)	(0.012)	(0.012)	(0.012)	(0.012)	(0.013)	(0.013)
Individual FE	No	Yes	No	Yes	No	Yes	No	Yes
N	4,473	4,473	4,473	4,473	4,473	4,473	4,482	4,482

Notes: The sample used is the early versus later treated sample. Each panel and column combination is from a separate regression. The outcomes are binary variables. Income quartiles are calculated from the year preceding the treatment period. Additional controls included are time fixed effects, sex, age groups (25-34, 35-44, 45-54, 55-64, 65-74, and > 74), log annual income, education, occupational class, and the grouped number of special medicine reimbursements (1, 2-3, and > 3). The control for sex is omitted from the regressions that control for individual fixed effects. The standard errors are clustered at the individual level and are shown in parentheses. * p < 0.1, ** p < 0.05, *** p < 0.01.

Table C5: Effect of diabetes on private dentist visits, by income quartile

Variable	Quartile 1	Quartile 1	Quartile 2	Quartile 2	Quartile 3	Quartile 3	Quartile 4	Quartile 4
Treatment	0.005		0.036		0.021		0.027	
	(0.016)		(0.023)		(0.025)		(0.027)	
After	−0.013	−0.003	−0.037*	−0.036*	−0.032	−0.037	0.059*	0.021
	(0.020)	(0.017)	(0.023)	(0.018)	(0.030)	(0.023)	(0.032)	(0.026)
Treatment×After	0.003	0.004	0.007	0.009	−0.011	−0.001	−0.019	−0.025
	(0.012)	(0.012)	(0.015)	(0.014)	(0.019)	(0.018)	(0.022)	(0.021)
Individual FE	No	Yes	No	Yes	No	Yes	No	Yes
N	4,473	4,473	4,473	4,473	4,473	4,473	4,482	4,482

Notes: The sample used is the early versus later treated sample. Each column is from a separate regression. The outcome is a binary variable. Income quartiles are calculated from the year preceding the treatment period. Additional controls included are time fixed effects, sex, age groups (25-34, 35-44, 45-54, 55-64, 65-74, and > 74), log annual income, education, occupational class, and the grouped number of special medicine reimbursements (1, 2-3, and > 3). The control for sex is omitted from the regressions that control for individual fixed effects. The standard errors are clustered at the individual level and are shown in parentheses. * $p < 0.1$, ** $p < 0.05$, *** $p < 0.01$.
